# Influence of perioperative blood pressure regulation on postoperative delirium in patients undergoing head and neck free flap reconstruction

**DOI:** 10.1186/s40001-023-01367-1

**Published:** 2023-09-22

**Authors:** Mark Ooms, Ruth Schooß, Philipp Winnand, Marius Heitzer, Frank Hölzle, Johannes Bickenbach, Annette Rieg, Ali Modabber

**Affiliations:** 1 Department of Oral and Maxillofacial Surgery, University Hospital RWTH Aachen, Pauwelsstraße 30, 52074 Aachen, Germany; 2 Department of Intensive Care Medicine, Uniklinik RWTH Aachen, Pauwelsstraße 30, 52074 Aachen, Germany; 3 Department of Anaesthesiology, Uniklinik RWTH Aachen, Pauwelsstraße 30, 52074 Aachen, Germany

**Keywords:** Postoperative delirium, Microvascular free flap, Head and neck reconstruction, Risk factor, Blood pressure regulation

## Abstract

**Background:**

Postoperative delirium (POD) is a serious complication in patients undergoing microvascular head and neck reconstruction. Whether intraoperative and postoperative blood pressure regulation are risk factors for POD remains unclear. This study aimed to highlight the relationships between intraoperative and postoperative blood pressure regulation and POD in microvascular head and neck reconstruction.

**Methods:**

Data from 433 patients who underwent microvascular head and neck reconstruction at our department of oral and maxillofacial surgery between 2011 and 2019 were retrospectively analyzed. The 55 patients with POD were matched with 55 patients without POD in terms of tracheotomy, flap type, and flap location, and the intraoperative and postoperative systolic and mean blood pressure values were compared between the two groups.

**Results:**

Patients with POD showed lower intraoperative and postoperative minimum mean arterial pressure (MAP) values than patients without POD (60.0 mmHg vs. 65.0 mmHg, *p* < 0.001; and 56.0 mmHg vs. 62.0 mmHg, *p* < 0.001; respectively). A lower intraoperative minimum MAP value was identified as predictor for POD (odds ratio [OR] 1.246, 95% confidence interval [CI] 1.057–1.472, *p* = 0.009). The cut-off value for intraoperative MAP for predicting POD was  ≤ 62.5 mmHg (area under the curve [AUC] 0.822, 95% CI 0.744–0.900, *p* < 0.001).

**Conclusions:**

Maintaining a stable intraoperative minimum MAP of  > 62.5 mmHg could help to reduce the incidence of POD in microvascular head and neck reconstruction.

## Introduction

Postoperative delirium (POD) is defined as a change in a patient’s mental status due to physical problems related to surgery and is characterized by an acute onset and fluctuating disturbances in consciousness, attention, and cognition [1, 2]. Although POD usually only lasts a few days, it remains a serious surgical complication that is associated with high morbidity and mortality, longer hospital stays, and, consequently, higher healthcare related costs [3].

POD is a common complication in patients who undergo head and neck surgery, with incidence rates ranging from 7.5 to 33.3% [4–7]. Many factors likely contribute to POD after head and neck surgery, but only a few have been clearly identified [8, 9]. These include older age, male gender, higher American Society of Anesthesiologists score (ASA), preoperative arterial hypertension, microvascular free flap reconstruction, tracheotomy, neck dissection, and blood transfusion [8, 9]. The identification and modification of further risk factors for POD may reduce the incidence of POD and improve patient outcomes [5, 10].

Intraoperative and postoperative blood pressure regulation may be additional risk factors for POD; systemic blood pressure is a major determinant of cerebral perfusion, and cerebral hypoxia due to cerebral hypoperfusion is involved in the pathophysiology of POD [11–14]. Under normal physiological conditions, systemic blood pressure is tightly regulated, and cerebral perfusion is maintained by cerebral autoregulation over a wide range of mean arterial blood pressure (MAP) [15]. Perioperatively, however, these mechanisms may be impaired, and fluctuations in blood pressure are commonly observed in patients who undergo surgical procedures [14, 16, 17].

Several studies have investigated the association between blood pressure regulation (i.e., absolute values or fluctuations of systolic blood pressure [SBP] and MAP) and POD, with conflicting results—associations have been both confirmed and denied [13, 17–23]. However, no study has yet examined the association between blood pressure regulation in head and neck surgery involving microvascular free flaps and POD; patients undergoing this procedure are at high risk for POD, and iatrogenic enhanced blood pressure regulation to maintain adequate free flap perfusion plays an important role in microvascular surgery [4, 8, 24]. It remains unclear whether intraoperative and postoperative blood pressure regulation are risk factors for POD in patients who undergo microvascular head and neck reconstruction and whether a threshold for minimum MAP can be determined to prevent POD.

The aim of this study was to investigate the relationships between intraoperative and postoperative blood pressure regulation and POD in patients undergoing microvascular head and neck reconstruction.

## Methods

### Study population

This study was approved by the local ethics committee of the Medical Faculty of RWTH Aachen University (EK 144-18) and all methods were in accordance with the relevant guidelines and regulations.

Data from 433 patients who underwent reconstruction in the head and neck region with microvascular free flaps in our department of oral and maxillofacial surgery between 2011 and 2019 were retrospectively analyzed. The patients were surgically treated for malignant or non-malignant diseases. The data were obtained from clinical notes and operation reports. Patients with neurologic diseases, psychiatric diseases, or substance use disorders (except for excessive alcohol consumption and smoking) were excluded from the study. Patients with incomplete or missing data were also excluded.

Further analysis was performed for all 55 patients with POD and 55 matched patients without POD with respect to surgical procedure parameters (i.e., transplant type, transplant location, and tracheotomy). The diagnoses for patients without POD and patients with POD, respectively, were adenocarcinoma (*n* = 2 and *n* = 0), ameloblastoma (*n* = 2 and *n* = 1), atrophies (*n* = 0 and *n* = 1), basal cell carcinoma (*n* = 0 and *n* = 2), medication-related osteonecrosis of the jaw (*n* = 0 and *n* = 1), melanoma (*n* = 0 and *n* = 1), Merkel cell carcinoma (*n* = 2 and *n* = 0), myxoma (*n* = 1 and *n* = 0), nerve sheath tumor (*n* = 0 and *n* = 1), osteomyelitis (*n* = 2 and *n* = 1), squamous cell carcinoma (*n* = 40 and *n* = 43), squamous cell carcinoma metastasis (*n* = 1 and *n* = 0), osteoradionecrosis of the jaw (*n* = 1 and *n* = 2), sarcoma (*n* = 2 and *n* = 2), salivary gland carcinoma (*n* = 1 and *n* = 0), and trauma (*n* = 1 and *n* = 0).

### Intraoperative and postoperative courses

All surgical procedures were performed under general anesthesia with intravenous or volatile narcotics, muscle relaxants, and opioids. Each patient’s MAP and SBP were monitored via an intraarterial catheter. Blood pressure was adjusted as needed using intravenous norepinephrine. The patients were intubated via tracheotomy tubes or oral tubes and had a central intravenous catheter. After surgery, the patients were admitted to the intensive care unit (ICU). Postoperative intensive care management included sedation and mechanical ventilation until at least the first postoperative morning. In addition, blood pressure was adapted to a target SBP of > 125 mmHg until the first postoperative morning. 5000 IU of heparin was administered subcutaneously three times daily for seven days. Enteral nutrition was administered via nasogastric tube until the patient was able to swallow. If POD occurred, the patients received psychoactive medications (clonidine, haloperidol, quetiapine, risperidone, pipamperone), and a psychiatrist was consulted if needed.

### Variables

Variables included demographic data (such as sex and age) and clinical data (such as body mass index (BMI), American Society of Anesthesiologists score (ASA), excessive alcohol consumption, smoking, diagnosis of preoperative arterial hypertension, preoperative antihypertensive medication, transplant type, transplant location, tracheotomy, operation time, blood transfusion, intraoperative SBP, intraoperative MAP, postoperative SBP, postoperative MAP, transplant revision, transplant success, length of hospital stay, length of ICU stay, and postoperative complications). In line with commonly used definitions, smoking was defined as a condition if the patients had smoked daily for a period of more than 6 months at the time of the preoperative anesthesia preparation interview, and excessive alcohol consumption was defined as a condition if the patients consumed more than 40 g of pure alcohol per day (for men) or more than 20 g of pure alcohol per day (for women) at the time of the preoperative anesthesia preparation interview [25, 26]. A diagnosis of preoperative arterial hypertension was recorded if the diagnosis was confirmed according to the discipline-specific guidelines. Only prescribed medications were defined as permanent medications. Transplant revision was defined as a surgical revision of the anastomosis with return to the operating room, and transplant success was defined as transplant survival until discharge from the hospital. Sepsis, systemic inflammatory response syndrome, acute renal failure, and patient death were defined as postoperative complications.

Intraoperative blood pressure values were obtained from the graphical representations of blood pressure values in the clinical records (measured with the IntelliVue X2 M3002A device [Philips Medizin Systeme Boeblingen GmbH, Boeblingen, Germany] by two investigators [mean value was used in case of discrepancy between investigators]), and postoperative blood pressure values were obtained from an electronic recording system (measured with the IntelliVue X2 M3002A device [Philips Medizin Systeme Boeblingen GmbH, Boeblingen, Germany] and recorded with the IntelliSpace Critical Care and Anesthesia ICCA data management system [Philips Medizin Systeme Boeblingen GmbH, Boeblingen, Germany]). Intraoperative blood pressure was measured invasively via an arterial catheter; postoperative blood pressure was measured invasively via an arterial catheter or non-invasively via a blood pressure cuff. Non-invasive blood pressure measurement data were used only when invasive blood pressure measurement data were not available. The patient’s SBPs and MAPs were recorded intraoperatively at 15-min intervals and postoperatively at 5-min intervals. The reference values for SBP and MAP were obtained from the routinely performed blood pressure measurement on the day before surgery or, if not available, calculated as the mean of all preoperative blood pressure measurements in the operation room before the induction of anesthesia. The time of anesthesia induction was defined as the time of administration of the induction medication. Intraoperative and postoperative SBP and MAP were determined as absolute minimum and maximum values. The deviations in SBP and MAP were calculated as the absolute difference between the maximum value and the reference value for upward deviation and as the absolute difference between the reference value and the minimum value for downward deviation. If the maximum value was lower than the reference value or the minimum value was higher than the reference value, the deviation was set to zero. All blood pressure values were analyzed for patients without POD until discharge from the ICU; all blood pressure values were analyzed for patients with POD until the occurrence of POD.

### Delirium

The diagnosis of POD was based on the Confusion Assessment Method (CAM) for ICU or by a validated chart review method, considering the words “delirium” and “delirious”, in line with the criteria of the Diagnostic and Statistical Manual of Mental Disorders (Fourth Edition) [1, 27–29]. All cases of delirium identified through chart review were validated by a second investigator. With regard to CAM, a diagnosis of POD was established when an acute onset and fluctuating course, inattention, and disorganized thinking or an altered level of consciousness were detected [21, 27]. This study did not distinguish between hyperactive, hypoactive, and mixed subtypes of POD.

### Statistical analysis

The patients were divided into those with POD and those without POD. The patients were also divided into two classes according to ASA (> 2 and ≤ 2). Descriptive analyses were performed separately for patients without POD and patients with POD, with continuous variables expressed as medians with interquartile ranges (IQRs) and categorical variables expressed as numbers with percentages (%). Testing for differences between patients with POD and patients without POD was performed using McNemartest for categorical data or Wilcoxon signed rank test for metric data. Multivariable conditional logistic regression analysis was performed for variables that showed significant differences between patients with and without POD. Receiver operator characteristics analyses were performed for intraoperative MAPs, and the theoretical optimal cut-off MAP values for the prediction of POD were determined by the calculation of the Youden index [30]. The diagnostic accuracy was analyzed by calculating the area under the curve [31]. *P* < 0.05 were considered statistically significant. No correction for multiple testing was performed. The statistical analysis was performed using SPSS version 26 (SPSS, IBM, New York, USA).

## Results

### Patient characteristics

The analyzed study population included 110 patients out of a total of 433 patients, 55 of whom had POD. All 55 patients with POD were matched with patients without POD in terms of transplant type, transplant location, and tracheotomy (Table 1). The groups differed in terms of age (*p* = 0.011). The groups did not differ in terms of sex (*p* = 0.486), ASA (*p* = 0.063), BMI (*p* = 0.206), excessive alcohol consumption (*p* = 0.307), smoking (*p* = 0.307), preoperative arterial hypertension (*p* = 1.000), preoperative antihypertensive medication (all *p* > 0.05), operation time (*p* = 0.580), or blood transfusion (*p* = 0.327). No difference was observed between the groups for transplant success (*p* = 1.000), with 1 (1.8%) transplant loss (1 anterolateral thigh flap) in the group of patients without POD and 2 (3.6%) transplant losses (1 radial forearm flap, 1 anterolateral thigh flap) in the group of patients with POD.

**Table 1:** Demographic and clinical characteristics of the study population

Variable	Non-POD (*n* = 55)	POD (*n* = 55)	*p*-value
Sex (*n*)			
Male	32 (58.2%)	37 (67.3%)	0.486
Female	23 (41.8%)	18 (32.7%)	
Age (years)	65.0 (20.0)	73.0 (16.0)	**0.011**
BMI (kg/m^2^)	25.3 (7.3)	24.3 (5.9)	0.206
ASA (*n*)			
1 + 2	37 (67.3%)	26 (47.3%)	0.063
3 + 4	18 (32.7%)	29 (52.7%)	
Excessive alcohol consumption (*n*)	11 (20.0%)	17 (30.9%)	0.307
Smoking (*n*)	19 (34.5%)	25 (45.5%)	0.307
Preoperative arterial hypertension (*n*)	28 (50.9%)	29 (52.7%)	1.000
Preoperative antihypertensive medication (*n*)			
ACE inhibitors	16 (29.1%)	10 (18.2%)	0.238
AT antagonists	6 (10.9%)	13 (23.6%)	0.118
ß-blockers	10 (18.2%)	18 (32.7%)	0.170
Calcium antagonists	4 (7.3%)	7 (12.7%)	0.549
Diuretics	8 (14.5%)	14 (25.5%)	0.263
Transplant type (*n*)			
Radial forearm flap	24 (43.6%)	24 (43.6%)	=
Anterolateral thigh flap	25 (45.5%)	25 (45.5%)	
Fibular flap	3 (5.5%)	3 (5.5%)	
Iliac crest flap	1 (1.8%)	1 (1.8%)	
Lower leg perforator flap	1 (1.8%)	1 (1.8%)	
Latissimus flap	1 (1.8%)	1 (1.8%)	
Transplant location (*n*)			
Floor of mouth	7 (12.7%)	7 (12.7%)	=
Tongue	7 (12.7%)	7 (12.7%)	
Mandibula	19 (34.5%)	19 (34.5%)	
Maxilla	8 (14.5%)	8 (14.5%)	
Soft palate	4 (7.3%)	4 (7.3%)	
Cheek	3 (5.5%)	3 (5.5%)	
Extraoral	7 (12.7%)	7 (12.7%)	
Tracheotomy (*n*)			
No	9 (16.4%)	9 (16.4%)	=
Yes	46 (83.6%)	46 (83.6%)	
Operation time (min)	581.0 (150.0)	611.0 (183.0)	0.580
Blood transfusion (*n*)			
No	36 (65.5%)	42 (76.4%)	0.327
Yes	19 (34.5%)	13 (23.6%)	
Transplant revision (*n*)			
No	52 (94.5%)	38 (69.1%)	** < 0.001**
Yes	3 (5.5%)	17 (30.9%)	
Transplant success (*n*)			
No	1 (1.8%)	2 (3.6%)	1.000
Yes	54 (98.2%)	53 (96.4%)	
Duration of hospital stay (days)	17 (10)	23 (22)	** < 0.001**
Duration of ICU stay (days)	2 (1)	7 (11)	** < 0.001**
Postoperative Complications (*n*)			
No	54 (98.2%)	36 (65.5%)	** < 0.001**
Yes	1 (1.8%)	19 (34.5%)	

Demographic and clinical parameters are indicated as numbers (with percentage) or median (with interquartile range) (separately described for the group of patients without POD (non-POD) and the group of patients with POD (POD)); *p*-values corresponding to testing for differences between the group of patients without POD and patients with POD with McNemar test (sex, ASA, excessive alcohol consumption, smoking, preoperative arterial hypertension, ACE inhibitors, AT antagonists, ß-blockers, diuretics, blood transfusion, calcium antagonists, transplant revision, transplant success, postoperative complications) and Wilcoxon signed rank test (age, BMI, operation time, duration of hospital stay, duration of ICU stay); significant p-values are bold; = patients without POD and patients with POD are matched

*POD* postoperative delirium, *BMI* body mass index, *ASA* American Society of Anesthesiologists score, *ICU* intensive care unit

Compared to patients without POD, patients with POD underwent more transplant revisions (*p* < 0.001), with 17 (30.9%) transplant revisions compared to 3 (5.5%) transplant revisions. Patients with POD also had a higher rate of complications (*p* < 0.001), with 19 (34.5%) patients with POD having complications compared to 1 (1.8%) patient without POD having complications, and longer hospital stays (*p* < 0.001), with a median of 23 (IQR 22) days compared to a median of 17 (IQR 10) days for those without POD. Furthermore, patients with POD had longer ICU stays (*p* < 0.001), with a median of 7 (IQR 11) days compared to a median of 2 (IQR 1) days for those without POD.

### Postoperative delirium

In the 55 patients with POD, the median time of POD onset was on the second postoperative day (IQR 3 days).

### Comparison of blood pressure values between groups

The intraoperative and postoperative blood pressure values for SBP and MAP differed between patients with POD and patients without POD (Table 2).

**Table 2:** Blood pressure values

Variable	*Non-POD (n* = *55)*	*POD (n* = *55)*	*p*-value
Preoperative reference blood pressure			
SBP (mmHg)	140.0 (27.0)	130.0 (29.0)	0.332
MAP (mmHg)	97.0 (20.0)	93.0 (16.0)	0.118
Intraoperative systolic blood pressure			
SBP maximum (mmHg)	158.0 (24.0)	160.0 (33.0)	0.335
SBP minimum (mmHg)	89.0 (10.0)	85.0 (13.0)	0.313
SBP upward deviation (mmHg)	16.0 (29.0)	30.0 (33.0)	0.081
SBP downward deviation (mmHg)	50.0 (33.0)	45.0 (25.0)	0.318
Intraoperative mean arterial pressure			
MAP maximum (mmHg)	110.0 (15.0)	110.0 (15.0)	0.962
MAP minimum (mmHg)	65.0 (5.0)	60.0 (10.0)	** < 0.001**
MAP upward deviation (mmHg)	7.0 (22.0)	15.0 (21.0)	0.053
MAP downward deviation (mmHg)	32.0 (19.0)	33.0 (21.0)	0.234
Postoperative systolic blood pressure			
SBP maximum (mmHg)	171.0 (29.0)	184.0 (33.0)	**0.005**
SBP minimum (mmHg)	92.0 (17.0)	78.0 (30.0)	**0.003**
SBP upward deviation (mmHg)	29.0 (40.0)	51.0 (46.0)	**0.010**
SBP downward deviation (mmHg)	47.0 (25.0)	53.0 (26.0)	0.215
Postoperative mean arterial pressure			
MAP maximum (mmHg)	111.0 (30.0)	115.0 (37.0)	0.301
MAP minimum (mmHg)	62.0 (10.0)	56.0 (11.0)	** < 0.001**
MAP upward deviation (mmHg)	20.0 (37.0)	18.0 (39.0)	0.114
MAP downward deviation (mmHg)	36.0 (18.0)	36.0 (16.0)	0.379

Parameters are indicated as median (with interquartile range) (separately described for the group of patients without POD (Non-POD) and the group of patients with POD (POD)); patients matched for transplant type, transplant location and tracheotomy; maximum and minimum values are absolute values, upward deviation values = maximum value minus reference value (negative values are set to 0), downward deviation values = reference value minus minimum value (negative values are set to 0); *p*-values corresponding to testing for differences between the groups of patients without POD (Non-POD) and patients with POD (POD) with Wilcoxon signed rank test; significant p-values are bold

*SBP* systolic blood pressure, *MAP* mean arterial blood pressure

**Fig. 1:**
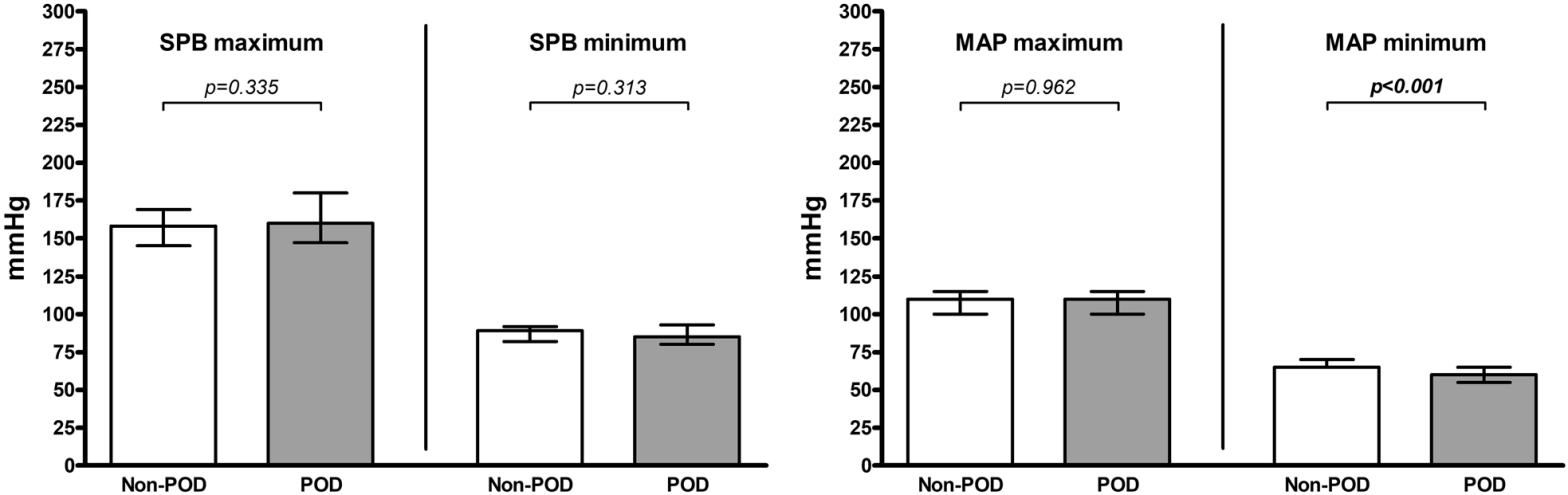
Intraoperative maximum and minimum blood pressure. Each column represents a median (with interquartile range) for intraoperative maximum and minimum blood pressure values for systolic blood pressure (SBP) (left) and mean arterial pressure (MAP) (right) separately for the group of patients without POD (Non-POD) and the patients with POD (POD); *p*-values corresponding to testing for differences with Wilcoxon signed rank test; significant *p*-values are bold

Patients with POD had a lower intraoperative minimum MAP than patients without POD (*p* < 0.001), with a median of 60.0 (IQR 10.0) mmHg compared to a median of 65.0 (IQR 5.0) mmHg (Fig. 1).

**Fig. 2:**
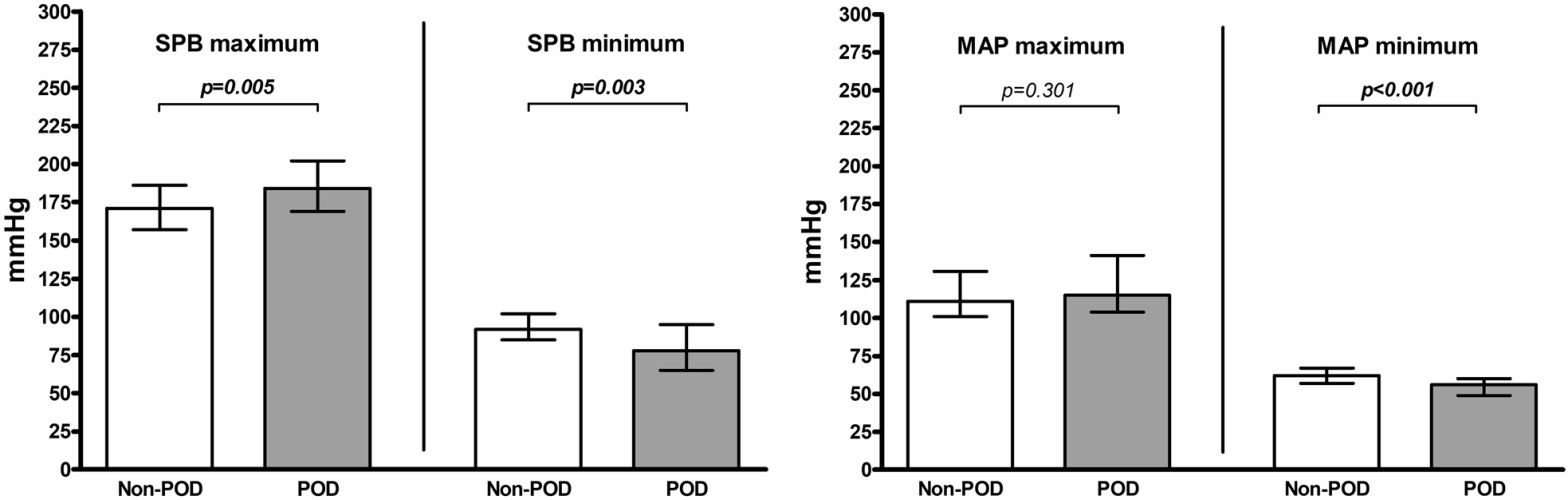
Postoperative maximum and minimum blood pressure. Each column represents a median (with interquartile range) for postoperative maximum and minimum blood pressure values for systolic blood pressure (SBP) (left) and mean arterial pressure (MAP) (right) separately for the group of patients without POD (Non-POD) and the patients with POD (POD); *p*-values corresponding to testing for differences with Wilcoxon signed rank test; significant *p*-values are bold

**Fig. 3:**
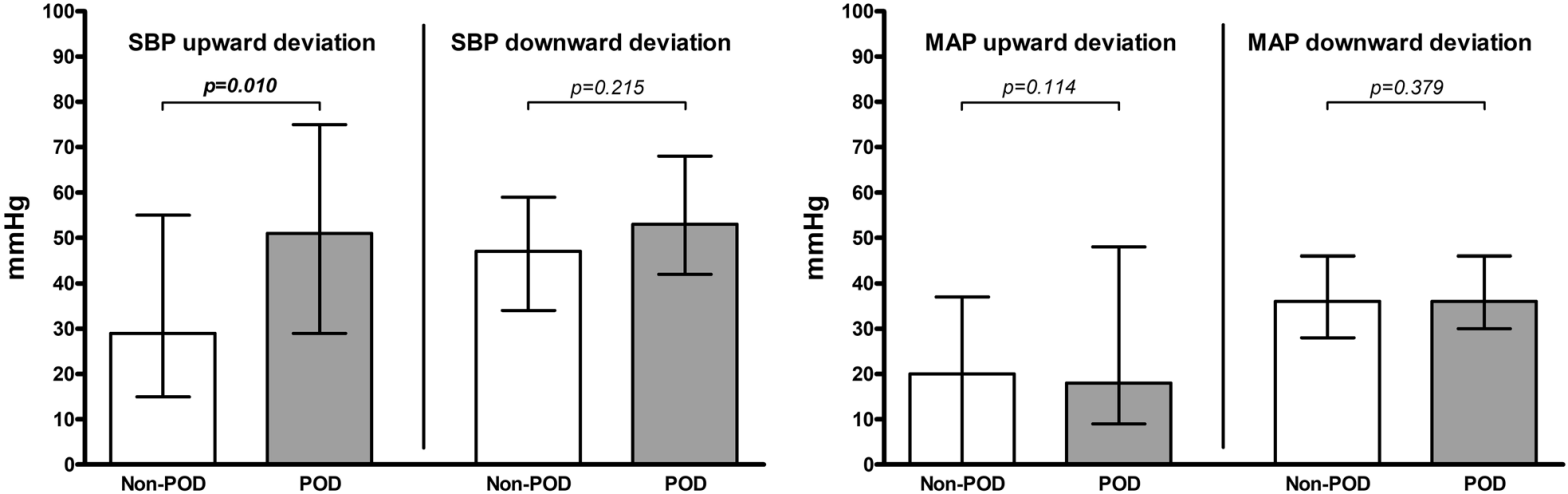
Postoperative upward and downward blood pressure deviation. Each column represents a median (with interquartile range) for upward and downward blood pressure deviation values for systolic blood pressure (SBP) (left) and mean arterial pressure (MAP) (right) separately for the group of patients without POD (Non-POD) and the patients with POD (POD); upward deviation values = maximum value minus reference value (negative values are set to 0), downward deviation values = reference value minus minimum value (negative values are set to 0); *p*-values corresponding to testing for differences with Wilcoxon signed rank test; significant *p*-values are bold

Patients with POD had a higher postoperative maximum SBP than patients without POD (*p* = 0.005), with a median of 184.0 (IQR 33.0) mmHg compared to a median of 171.0 (IQR 29.0) mmHg (Fig. 2). Patients with POD had a lower postoperative minimum SBP than patients without POD (*p* = 0.003), with a median of 78.0 (IQR 30.0) mmHg compared to a median of 92.0 (IQR 17.0) mmHg (Fig. 2). Patients with POD had a lower postoperative minimum MAP than patients without POD (*p* < 0.001), with a median of 56.0 (IQR 11.0) mmHg compared to a median of 62.0 (IQR 10.0) mmHg (Fig. 2). In addition, patients with POD had a higher upward postoperative SBP deviation than patients without POD (*p* = 0.010), with a median of 51.0 (IQR 46.0) mmHg compared to a median of 29.0 (IQR 40.0) mmHg (Fig. 3).

### Predictors for postoperative delirium

The multivariable regression analysis identified intraoperative minimum MAP (odds ratio [OR] 1.246, 95% confidence interval [CI] 1.057–1.472), *p* < 0.001) as a predictor for POD (Table 3). Patients with lower intraoperative minimum MAPs were more likely to develop POD. Receiver operating characteristics analysis for intraoperative MAP (area under the curve [AUC] 0.822, 95% CI 0.744–0.900, *p* < 0.001) determined that the theoretical optimal cut-off value for predicting POD was ≤ 62.5 mmHg (sensitivity 0.727; specificity 0.800).

**Table 3:** Regression analysis

Variable	Comparison	Odds ratio (95% CI)	*p*
Intraoperative MAP minimum*	mmHg	1.246 (1.057–1.472)	**0.009**
Postoperative SBP maximum	mmHg	1.018 (0.971–1.068)	0.456
Postoperative SBP minimum*	mmHg	1.017 (0.967–1.070)	0.496
Postoperative SBP upward deviation	mmHg	0.999 (0.960–1.040)	0.978
Postoperative MAP minimum*	mmHg	1.116 (0.981–1.270)	0.095
Age	years	1.068 (0.986–1.156)	0.106
Transplant revision	No vs. Yes	1.682 (0.147–19.285)	0.676

Odds ratios (with 95% Confidence interval) and p-values are corresponding to multivariable conditional logistic regression analysis; *odds ratios and confidence intervals are inverted and apply to the decrease of the predictor variable by one unit; significant *p*-values are bold

*SBP* systolic blood pressure, *MAP* mean arterial blood pressure

## Discussion

The aim of this study was to investigate the potential roles of intraoperative and postoperative systemic blood pressure regulation as risk factors for POD in patients undergoing head and neck free flap reconstruction. The presumed multifactorial etiology of POD makes it likely that multiple risk factors interact, and although several risk factors have been identified in patients undergoing head and neck free flap reconstruction, data on systemic blood pressure regulation are lacking; findings to optimize intraoperative and postoperative blood pressure regulation would be directly applicable [9, 21, 32].

The potential roles of intraoperative and postoperative systemic blood pressure regulation as risk factors for POD are related to the pathophysiological mechanisms beyond POD, including cerebral hypoxia due to cerebral hypoperfusion [11–14]. Hence, inadequate intraoperative and postoperative blood pressure regulation could be risk factors for POD, as systemic blood pressure is a major determinant of cerebral perfusion [13]. Cerebral perfusion is regulated by several mechanisms to balance cerebral metabolic demand and supply [33, 34]. One of these mechanisms is cerebral autoregulation, which, under normal physiological conditions, maintains stable cerebral perfusion over a wide range of MAPs (double diastolic blood pressure plus systolic blood pressure divided by three), with 60 mmHg as the lower threshold and 160 mmHg as the upper threshold [35, 36]. The lowering of systemic blood pressure is a common side effect of general anesthesia in patients undergoing major surgery due to the vasodilatory and cardio-depressive effects of anesthetics [14, 16, 17]. It is also performed intentionally, as low blood pressure improves the visibility of the surgical field and reduces blood loss [37]. Given the current monitoring capabilities that allow for the rapid detection and treatment of hemodynamic abnormalities, the regulation of intraoperative and postoperative systemic blood pressure with avoidance of hypotension and hypertension could be a potential area of intervention to prevent POD [14, 18].

In the absence of uniform definitions of hypotension and hypertension, blood pressure values for MAP and SBP in this study were analyzed as metric variables without categorization based on predefined cut-off values [14, 16, 38]. Patients with POD were matched with patients without POD for previously identified risk factors for POD in head and neck surgery, such as transplant type, transplant location, and tracheotomy, to mitigate the potential confounding effects of these factors [8]. To include all patients with POD and to avoid the reduction of the study population, matching for other known risk factors was not performed. In this regard, it should be noted that the groups differed only with respect to age as previously identified risk factor for POD in head and neck surgery, and age was included as an independent variable in the multiple regression analysis.

This study identified a lower intraoperative minimum MAP as a predictor for POD. This is consistent with the findings of two previous studies, which showed that intraoperative MAPs below 65 mmHg and 60 mmHg, respectively, were associated with POD [13, 19]. Similarly, other studies have reported an association between the duration of intraoperative minimum MAP below 60 mmHg and POD [17, 22]. Moreover, one study showed that intraoperative minimum MAP was an independent predictor for POD and that higher intraoperative MAPs had a protective effect on the occurrence of POD [23]. Regarding blood pressure fluctuation, patients with and without POD showed differences in postoperative SBP deviation to higher values in this study’s univariable analysis, but these differences could not be identified as predictive factors for POD in the multivariable analysis. The findings of the present study are in line with those from another study, in which an intraoperative MAP of < 55 mmHg (but not MAP fluctuations) was associated with POD [39]. However, the results of other studies have differed; it has been shown that an intraoperative MAP of even < 50 mmHg was not associated with POD [21]. The discrepancies in results may be due to differences in surgical procedures between studies, as different risk factors interact depending on the surgical procedure. In general, there is a lack of comparable data from studies including only patients undergoing microvascular free flap reconstruction of the head and neck region. Interestingly, although postoperative minimum MAPs were lower in patients with POD compared to patients without POD in univariable testing, postoperative minimum MAPs only tended to be a predictor for POD in multivariable testing.

The associations between lower intraoperative minimum MAPs and POD observed in this study may be explained by lower cerebral perfusion due to lower MAP despite cerebral autoregulation according to the proposed pathophysiological mechanism of cerebral hypoxia for POD [11–14]. Interestingly, based on the data from this study, the calculated optimal cut-off values for predicting POD were ≤ 62.5 mmHg for the intraoperative minimum MAP, which is close to the lower limit of cerebral autoregulation of approximately 60 mmHg. With regard to the calculated optimal cut-off value for predicting POD at values around 60 mmHg, it should be taken into account that cerebral autoregulation thresholds are only theoretical values and are likely to vary across individuals, with the lower MAP threshold varying between 53 and 113 mmHg [15]. This may be related to comorbidities, such as chronic arterial hypertension, which increases the lower MAP threshold [40]. However, the preoperative diagnosis of arterial hypertension did not differ between patients with and without POD in this study. Although the cut-off value for minimum MAP can only serve as an approximate orientation regarding the sensitivity and specificity for predicting POD, it may be useful for the management of intraoperative blood pressure [18].

This study has some limitations that require consideration. These include a retrospective chart review with limited documentation quality, a lack of assessment of preoperative cognitive impairments, and the absence of mental health professionals for the diagnosis of POD. Nevertheless, the ICU-CAM tool used in this study has shown a strong correlation with expert opinion [41]. Furthermore, patients with neurologic comorbidities, psychiatric comorbidities, and substance use disorders (except for excessive alcohol consumption and smoking) were excluded to mitigate the effects of a lack of preoperative assessment of cognitive impairments. In general, it should be noted that several mechanisms besides cerebral autoregulation, such as cardiac output, neurovascular coupling related to cerebral metabolic activity, and cerebrovascular reactivity related to carbon dioxide and oxygen blood concentration, influence cerebral perfusion [34]. Therefore, the monitoring of cerebral perfusion may be a more appropriate approach for evaluating the relationship between blood pressure regulation and POD. Additionally, limitations in the accuracy of the blood pressure values obtained from graphical representations cannot be excluded; however, the values were obtained by two investigators for each patient, with the mean value used in case of discrepancy between investigators. Furthermore, it cannot be excluded that variables other than that measured have an influence on the development of POD, such as the cause of intraoperative low blood pressure (intentional or not) or the number of blood transfusions.

In this study, a matched-pair analysis was used to study a particular patient group that has not been specified in previous studies. The findings of the present study show that lower intraoperative MAPs are a predictor for POD. These findings may contribute to the further optimization of patient care (through the reduction of POD) via the maintenance of intraoperative MAPs above 62.5 mmHg. Further studies are needed to confirm these results and to investigate the potential benefits of an intervention aimed at minimizing excessively low MAP values.

## Conclusion

This study found that low intraoperative minimum MAPs are modifiable risk factors for POD in patients undergoing head and neck surgery with microvascular free flap reconstruction. This knowledge has implications for both the further prevention and the treatment of POD. Maintaining an intraoperative minimum MAP of > 62.5 mmHg may reduce the incidence of POD, and the close monitoring of patients with lower intraoperative minimum MAPs may help clinicians to initiate treatment earlier, reducing the severity of POD.

## Data Availability

The datasets used during the current study are under further analysis and are available from the corresponding author on reasonable request.

## Abbreviations

POD: Postoperative delirium
MAP: Mean arterial blood pressure
SBP: Systolic blood pressure
ICU: Intensive care unit
BMI: Body mass index
ASA: American Society of Anesthesiologists score
CAM: Confusion Assessment Method
